# Health behaviours of patients with affective disorders: a cross-sectional study

**DOI:** 10.1186/s12888-023-05056-5

**Published:** 2023-08-04

**Authors:** Krystyna Górna, Renata Szpalik, Janusz K. Rybakowski, Krystyna Jaracz

**Affiliations:** 1 Department of Psychiatric Nursing, Poznan University of Medical Sciences, Rokietnicka 2A, Poznań, 60-806 Poland; 2 Department of Adult Psychiatry, Poznan University of Medical Sciences, Szpitalna 27/33, Poznań, 60-572 Poland; 3 Department of Neurological Nursing, Poznan University of Medical Sciences, Rokietnicka 2A, Poznań, 60-806 Poland

**Keywords:** Affective disorders, Health behaviours, Lifestyle

## Abstract

**Background:**

Severe mental disorders, including affective disorders (AD), are associated with high rates of physical illnesses that lead to premature patient death. Excess somatic comorbidity may be partially explained by lifestyle factors. This study aimed to investigate the health behaviours (HBs) of patients with AD in comparison to the HBs of patients with type 2 diabetes (T2D) and healthy controls (HCs) and to examine associations among HBs and sociodemographic and clinical factors, subjective quality of life and health status, and health locus of control.

**Methods:**

The sample consisted of 108 patients with AD, including 60 with bipolar disorder (BP) and 48 with unipolar disorder (UAD). Analyses included comparisons with a subgroup of AD individuals, patients with T2D and HCs matched in age and sex. The Health Behaviour Inventory was used to evaluate the overall levels of HBs and 4 HB categories. To identify independent determinants of health behaviours, a multivariate linear regression analysis was performed with factors identified as significant in bivariate analyses.

**Results:**

Most AD patients had a low level of HBs (40%), followed by moderate (35%) and high levels (25%), and there were no significant differences in HBs between the BP and UAD groups. Compared with the T2D and HC groups, the AD group had a significantly lower level of overall HBs and lower levels of HBs in one of the categories. Independent predictors of overall HBs were quality of life (β = 0.28, p < 0.001), age (β = 0.27, p = 0.002), and depressive symptoms (β = 0.23, p = 0.008). A total of 30% of the variance in HBs was explained.

**Conclusions:**

These findings emphasise the need for a systematic assessment of single and multiple health behaviours to provide better care for patients with AD and reduce the potential adverse effects of an unhealthy lifestyle.

## Background

Affective disorders (AD; mood disorders), including depression and bipolar disorder (BP), are one of the most critical global health [1, 2] and socioeconomic problems [3].

They are among the leading causes of disability worldwide [4–7]. The 2017 Global Burden of Disease (GBD) study estimated that among persons with depressive disorders, the number of healthy life years lost (according to the WHO disability-adjusted life years (DALYs) index) increased by 52.3%, from 28.30 million in 1990 to 43.10 million in 2017; among those with bipolar disorder, the DALYs increased by 54.4%, from 6.02 million in 1990 to 9.29 million in 2017 [8]. Factors responsible for the high health-related global burdens of these disorders include their prevalence, symptom severity, chronicity, functional impairment and corresponding limitations in multiple aspects of a person’s life [9].

Affective disorders, like other severe mental illnesses, are associated with premature mortality [10, 11]. Patients with these disorders die approximately 10–20 years earlier than the general population [12], with a reported death rate of 14.3% (approximately 8 million per year), of which approximately 67% of deaths are due to natural causes and only 17.5% of deaths are due to unnatural causes, such as suicide, homicide and accidents [13]. Among natural causes, comorbid somatic diseases are most frequently mentioned [14, 15]. Individuals with AD have a higher prevalence rates of cardiovascular, respiratory, and endocrine diseases; obesity; hypertension; hyperlipidaemia; type 2 diabetes; and other diseases than the general population [15]. Among the causes of poor somatic health in people with severe mental illness are the symptoms of mental disorders themselves [16], the side effects of medication [17, 18], impaired access to health services, worse quality of these services [15] and lifestyle factors manifesting in behaviours leading to direct or indirect and more or less distant adverse health consequences [12, 19]. Generally, health behaviours undertaken by an individual may be defined as “behavior patterns, actions and habits that relate to health maintenance, to health restoration and to health improvement” [20].

According to studies on various health behaviours, compared to normative populations or control groups, patients with severe mental disorders are significantly more likely to be characterised by lower consumption of healthy foods, lower levels of physical activity, and more frequent smoking and alcohol consumption, among other behaviours [21–23]. Most studies on health behaviours of AD patients conducted thus far have examined single, isolated behaviours, and less have analysed health behaviours holistically as a set of behaviours that constitute a broader lifestyle component [21, 24, 25], which, according to Lalonde’s classic “health fields” paradigm, determines more than 50% of peoples’ health [26]. In addition, previously published research on the health behaviours of patients with severe mental disorders lacked empirical data from Poland. Therefore, the present work may contribute to prior knowledge and supplement the research literature on the topic.

The present study aimed to explore the health behaviours of patients with affective disorders, with a specific focus on (1) evaluating the overall level of health behaviours and levels of specific categories of these behaviours; (2) comparing the health behaviours of patients with affective disorders with those of patients with a chronic somatic illness (type 2 diabetes) and healthy individuals; and (3) analysing relationships of health behaviours with sociodemographic and clinical variables as well as subject factors (subjective quality of life and health status, and health locus of control).

The selection of potential factors that may determine health behaviours, such as sociodemographic variables, mental health (i.e., depression), health locus of control, self-rated health and quality of life, was determined based upon a review of the literature [27–31].

## Methods

### Study design and participants

This cross-sectional study was conducted from January 2017 to December 2018. The study sample consisted of a group of patients with affective disorders (AD) hospitalised at the Department of Adult Psychiatry at the University of Medical Sciences and two comparison groups, including patients with type 2 diabetes (T2D) treated at the Internal Medicine and Diabetology Department (Group 1) and healthy subjects registered at a GP outpatient clinic (Group 2). Individuals in the comparison groups were recruited via collaboration with the physicians providing their medical care based on the inclusion criteria (listed below in this section).

Diabetic patients were included as one of the comparison groups because diabetes and affective disorders have some common features, such as chronicity, complex and multifactorial aetiology, symptomatic treatment, substantial impact on a patient’s quality of life and the importance of health behaviours for general health and treatment outcome. Nevertheless, these two conditions differ significantly in terms of illness specificity and clinical symptoms.

The AD group consisted of 108 patients, including 60 with bipolar disorder (BP) and 48 with unipolar disorder (UAD). Diagnosis was made by psychiatrists according to ICD-10 criteria, and the study was conducted during the period of improvement in their mental status before their discharge from the hospital. Inclusion criteria were an age of 18 years or above, ability to answer the research questions, and written consent to participate in the study after being informed about the purpose and nature of the study.

The comparison groups were matched in terms of age and to a subgroup of 65 individuals in the AD group. *Propensity matching analysis* (PMA) using the nearest neighbour method without return was used to select the groups.

The T2D group included 67 patients without diagnosed or documented mental illnesses or psychopharmacological treatment. All patients received antidiabetic therapy. Among them, 40 (59.7%) individuals were exclusively prescribed insulin (long-acting and/or rapid-acting), 11 (16.4%) individuals solely took oral antidiabetic drugs, and 16 patients (23.9%) received combination therapy involving both oral drugs and insulin.

Additionally, patients with T2D received antihypertensive medication (n = 51; 76.1%), cholesterol-lowering drugs (n = 39; 58.2%), and cardiovascular medications (n = 39; 46.3%). The duration of the disease in this group ranged between 1 and 45 years (mean = 12.7 ± 8.99).

The healthy control group consisted of 46 individuals without diagnosed severe illnesses, including mental or somatic conditions, and not receiving psychopharmacological treatment.

### Assessments

This study used a set of 5 standardised research tools:

1. The Health Behaviour Inventory (HBI) by Juczyński [32] was used for the assessment of 24 health-related behaviours, scored on a scale from 1 to 5. The HBI includes an overall health behaviour index (HB, score range of 24–120) and four behaviour categories, each of which contains 6 items: healthy eating (HE), preventive behaviours (PB), positive mental attitude (PMA) and health practices (HP). Scores on the above categories are presented as the sum of the scores of each item (score range of 6–30). The overall HB score was transformed into standardised units according to sten scores (range: 1 to 10 points), where scores of 1–4 indicate a low level of health behaviour, scores of 5–6 represent a moderate level of health behaviour and scores of 7–10 reflect a high level of health behaviour [32]. The HBI has been used in several studies and has demonstrated acceptable reliability [32]. In the present study, McDonald’s ω and Cronbach’s α for the total scale were 0.86 and 0.86, respectively, indicating satisfactory values; for the subscales, these values ranged between 0.86 and 0.71, demonstrating acceptable internal consistency, except for the health practices subscale, where the coefficients were lower (0.49 and 0.44, respectively).
2. The Polish version of the Beck Depression Inventory (BDI) was used to assess the severity of depressive symptoms. The BDI contains 21 items with possible sum scores ranging from 0 to 63, where scores of 0–11, 12–26, 27–49, and 50–63 indicate no depression, mild depression, moderate depression, and severe or very severe depression, respectively [33]. The BDI has good reliability [34]. In the present sample, the ω and α values were 0.94 and 0.93, respectively.
3. The Global Assessment Functioning scale (GAF) was used to assess the overall level of functioning [35, 36]; this scale has ten 10-point subscales. The total score ranges from 1 to 100, with a lower score indicating a poorer level of functioning. A GAF score below 61 denotes moderate, severe or very severe impairment in psychosocial functioning.
4. Two general questions derived from the Polish version of the WHOQoL-Bref questionnaire were used for subjective assessment of the quality of life and health status, with questions scored on a scale from 1 to 5 [37]. Higher scores correspond to better status. For the purposes of this study, scores of 1–3 were defined as a low result, and scores of 4–5 were defined as a high result.
5. The Multidimensional Health Locus of Control scale (MHLC-B) [32, 38] was used to assess the intensity of an individual’s beliefs about the extent to which they are in control of their health. The MHLC includes three dimensions: internal locus of control (I), powerful others (P) and chance (C). On each of these dimensions, the respondent can score between 6 and 36 points. The higher the score is, the stronger the belief that a given factor affects their health. Satisfactory internal consistency was previously reported [32]. In the present study, the reliability coefficients for the total scale and subscales were also favourable: for the total scale, ω and α = 0.82; for the I and P subscales, ω and α = 0.74; for the C subscale, ω and α = 0.72.

A self-report questionnaire of sociodemographic and clinical data was also administered in the study.

### Statistical analyses

Means, standard deviations, ranges, frequencies, and percentages were used for statistical description. For all continuous variables, the conformity of their distribution with the normal distribution was checked using the Shapiro‒Wilk test. For correlation analyses, Pearson’s (rp) or Spearman’s correlation (rs) coefficient was used, depending on the type of variable and whether it was normally distributed. Appropriate parametric or nonparametric tests (Student’s *t* test, Mann‒Whitney *U* test, one-way ANOVA or Kruskal‒Wallis test) were used for group comparisons. When comparisons among the three study groups indicated statistically significant differences, Tukey’s post hoc test or a post hoc Dunn test of multiple comparisons (two-tailed) were used. Relationships between qualitative variables were explored using the chi-square test or Fischer’s exact test and the test for two proportions. Multivariate analysis was performed using stepwise forward linear regression analysis. The significance level was set at p < 0.05. The analysis was performed using Statistica 13 (StatSoft Inc.).

## Results

### Sociodemographic and clinical characteristics of persons with AD

A total of 108 patients with AD (33% male, 67% female), aged 18–89 (mean 47) years, participated in the study. More than half of the patients were unmarried (54%). The majority lived with other persons (86%), lived in urban or suburban areas (76%), had secondary education (45%) or higher education (30%), and did not work in a professional sector (65%).

Of the subjects, 56% suffered from BP and 44% from UAD. All patients received appropriate psychopharmacological treatment, including mood-stabilising drugs, antipsychotics, and antidepressants.

The mean duration of illness was approximately 12 (range 0.5–48) years. Of the patients, 69% had somatic diseases, mainly hypertension (25%), metabolic disorders (20%) and gastrointestinal diseases (18%). The average BDI score indicated mild severity of depressive symptoms, with 62% of patients showing no or mild symptoms according to the adopted score ranges. The average level of functioning, according to the GAF, indicated minor or moderate impairment in global functioning. None of the patients demonstrated severe or very severe psychosocial impairment. A total of 39% attended psychoeducation (Table 1).

**Table 1:** Sociodemographic and clinical characteristics of patients with affective disorders (n = 108)

Characteristics	n (%) / mean (± SD)
Age (years)	47.55 (*±* 15.13)
Gender	
male	36 (33)
female	72 (67)
Marital status	
in a permanent relationship	50 (46)
not in a relationship	58 (54)
Living arrangement	
alone	15 (14)
with others	93 (86)
Place of residence	
village	26 (24)
urban or suburban	82 (76)
Education	
primary/occupational	27 (25)
secondary	49 (45)
higher	32 (30)
Source of livelihood	
working	38 (35)
not working	70 (65)
Diagnosis (according to ICD-10)	
bipolar affective disorder	60 (56)
unipolar affective disorder	48 (44)
Duration of disease (years)	11.80 (± 10.98)
Somatic comorbidities	74 (69)
yes	74 (69)
no	34 (31)
Severity of depressive symptoms (according to BDI)	22.51 (± 13.49)
Global functioning (according to GAF)	59.61 (± 4.56)
Participation in psychoeducation: yes	
yes	42 (39)
no	66 (61)

Annotations: n, number; SD, standard deviation

### Health behaviours of patients with AD

The mean overall HB score was 79.21 ± 15.62 points, slightly above the midpoint of the scale range (72 points). For the HB sten scores, most were low (40%), followed by moderate (35%) and high scores (25%). Of the four HB categories, the highest scores were for preventive behaviour (PB), and the lowest scores were for positive mental attitude (PMA). There were no significant differences in the mean level of the HB index between subjects with BP and UAD (Table 2).

**Table 2:** Health behaviours of patients with affective disorders (n = 108). Comparative analysis among unipolar bipolar (n = 60) and bipolar (n = 48) groups

Variable	Affective Disorders (total sample) (n = 108)	Groups according to diagnosis		*p*
		Bipolar disorder (n = 60)	Unipolar disorder (n = 48)	
	n (%) / mean (± SD)/median (Q1; Q3)			
Overall index (HBs)	79.21 (± 15.62)	79.77 (± 15.15)	78.52 (± 16.31)	0.682^a^
HB sten scores (ranges)				
low (1–4)	43 (40)	24 (40)	19 (40)	0.584^b^
moderate (5–6)	38 (35)	19 (32)	19 (40)	
high (7–10)	27 (25)	17 (28)	10 (21)	
Health behaviour categories				
good eating habits (EH)	19.76 (± 5.91)	19.60 (± 5.99)	19.96 (± 5.86)	0.756^a^
preventive behaviour (PB)	20.37 (± 5.25)	20.67 (± 5.28)	20.00 (± 5.24)	0.514^a^
positive mental attitude (PMA)	18.98 (± 5.17) 19.5 (15; 23)	19.32 (± 4.94) 21 (15; 23)	18.56 (± 5.47) 18 (15;22)	0.306^c^
health practices (HP)	20.10 (± 4.15) 20 (18; 23)	20.18 (± 4.21) 20 (18; 23)	20.00 (± 4.11) 20 (17; 22)	0.672^c^

Annotations: SD, standard deviation; HBs, health behaviours;

^a^ Student *t* test, ^b^ Chi-square test, ^c^ Mann-Whitney *U* test

The two groups also did not differ in most sociodemographic and clinical characteristics. The only differences were in residential location and duration of illness. Compared to those with UAD, individuals with BP were more likely to live in a city (51% vs. 31%, *p* = 0.014) and to be ill for a longer period (mean 14.49 ± 15.76 vs. 8.23 ± 14.33, *p* < 0.001). Given the absence of significant differences in health behaviours and most sociodemographic characteristics, the two groups were pooled together for further analyses.

### Health behaviours of patients with AD compared to health behaviours of patients with T2D and healthy individuals

Individuals with AD had significantly lower mean HB scores than the comparison groups (T2D, *p* = 0.0005; HC, *p* = 0.0197). Additionally, patients with AD had significantly lower HB scores than those in the comparison groups (T2D, *p* = 0.0039; HC, *p* = 0.0109) and significantly fewer high scores than T2D patients (*p* = 0.0009). Of the four HB categories, the AD group had a significantly lower mean score in the positive mental attitude category (PMA) relative to the comparison groups (T2D, *p* < 0.0001; HC, *p* = 0.0114) (Table 3). The analysis of individual items in this subscale revealed that the most significant differences occurred in responses to statements related to the frequency of positive thinking, avoiding emotions (such as anger and anxiety), and maintaining good relationships within the family. Responses of “almost always” and “often” were given by 30.7%, 32.3%, and 50.7% of AD patients, respectively. In the T2D group, these percentages were 80.6%, 65.7%, and 79.1%, while in the HC group, they were 71.7%, 52.2%, and 70%.

**Table 3:** Health behaviours of patients with affective disorders compared to health behaviours of patients with type 2 diabetes and healthy individuals

Health behaviours	Study groups			
	Affective disorders n = 65	Diabetes type 2 n = 67	Healthy n = 46	*p*
	n (%) / mean (± SD)			
Gender: female	41 (63)	34 (51)	27 (59)	0.345^e^
Age	55.51 (± 12.87) 56 (50; 60)	58.18 (± 5.95) 59 (54; 62)	55.09 (± 10.97) 53 (44; 64)	0.059^d^
Overall index (HBs)	79.20 (± 12.87)^a^	88.09 (± 13.45)^b^	86.74 (± 14.23)^b^	< 0.001^c^
HB sten scores (ranges)				
low (1–4 stens)	24 (37)	10 (15)	7 (15)	0.003^e^
moderate (5–6 stens)	26 (40)	23 (34)	21 (46)	
high (7–10 stens)	15 (23)	34 (51)	18 (39)	
Health behaviour categories				
good eating habits (EH)	19.43 (± 5.64) 20 (16; 24)	21.33 (± 4.72) 21 (19; 25)	21.30 (± 4.63) 21 (19; 25)	0.079^d^
preventive behaviour (PB)	20.69 (± 4.45)	22.04 (± 4.85)	22.37 (± 4.38)	0.111^c^
positive mental attitude (PMA)	19.11 (± 4.37)^a^ 21 (16; 22)	23.25 (± 3.73)^b^ 24 (16; 22)	22.00 (± 3.72)^b^ 22 (19;22)	< 0.001^d^
health practices (HP)	19.97 (± 4.00) 20 (18; 23)	21.46 (± 4.80) 22 (18; 25)	21.07 (± 4.47) 21.5 (18; 23)	0.131^d^

Annotations: Means not sharing subscripts (a, b) differ at *p* < 0.05

^c^ ANOVA *F* test, ^d^ Kruscal-Wallis *H* test, ^e^ Chi-square test

There were no significant group differences in the remaining three health behaviour categories; nonetheless, a trend towards significance was noted in the EH subscale (p = 0.07) (Table 3). Analysis of the items within the EH subscale showed that the greatest differences were in responses to statements regarding whole-grain bread consumption and avoidance of preserved food. The distributions of responses “almost always” and “often” were as follows: in the AD group, 44.6% and 39.8%, respectively; in T2D group, 61.2% and 57.7%.

### Relationship of health behaviours of patients with AD with their sociodemographic, clinical and subjective characteristics

The bivariate analyses of the relationship of health behaviours (according to the overall HB index) with potential determinants of these behaviours included sociodemographic, clinical and subject variables (subjective assessment of quality of life and health, health locus of control).

The mean HB index showed significant positive correlations with patients’ age (r_s_ = 0.30, p = 0.002) and their level of overall functioning according to the GAF score (r_s_ = 0.25, p = 0.011) and a negative correlation with the severity of depressive symptoms according to the BDI score (r_s_ = -0.34, p < 0.001). Furthermore, there were significant correlations between the mean HB index and subjective assessment of quality of life (low score, mean = 74.36 ± 14.48 points vs. high score, mean = 86.27 ± 14.61 points; p < 0.001) and health status (low score, mean = 77.33 ± 14.59 points vs. high score, mean = 87.00 ± 17.62 points; p = 0.010). Additionally, there was a significant negative correlation of the HB index with the health control locus in the dimension of powerful others (r_s_ = 0.21, p = 0.032). Other variables did not show significant relationships with the overall HB index (Table 4).

**Table 4:** Bivariate analyses of the relationships between health behaviours of patients with affective disorders according to the overall HBs index and sociodemographic, clinical and subject factors (n = 108)

Factors	Correlation coefficient *Rs*	*p*
Socio-demographic factors		
age (years)	0.30	0.002
gender		0.976
marital status		0.178
living arrangement		0.983
place of residence		0.648
education		0.137
source of livelihood		0.433
use of psycho-education		0.058
Clinical factors		
duration of illness (years)	0.06	0.510
somatic comorbidities		0.619
overall functioning (according to GAF)	0.25	0.011
severity of depressive symptoms (according to BDI)	-0.34	< 0.001
Subject factors		
subjective assessment of quality of life and health status (according to WHOQoL-Bref)		
quality of life		< 0.001
health status		0.010
dimensions of the health control locus (according to MHLC-B)		
Internal	0.16	0.098
Powerful others	0.21	0.032
Chance	-0.02	0.994

Annotations: *Rs* Spearman correlation coefficient

To identify independent determinants of health behaviours, a multivariate linear regression analysis was performed in which factors identified as significant in the bivariate analyses were included. Poor quality of life (β = 0.28), advanced age (β = 0.27) and lower severity of depressive symptoms (β = 0.23) were found to predict higher level of health behaviour. Together, these factors and the nonsignificant health locus of control - powerful others variable explained 30% of the total variance in the HB index (Table 5).

**Table 5:** Independent determinants of health behaviours of patients with affective disorders according to the overall HB index (n = 108)

Factors	Overall HBs indicator			
	*R* ^*2*^	*β*	*F*	*p*
	30%		11.10	< 0.001
subjective assessment of quality of life (according to WHOQoL-Bref)^a^		0.28		0.001
age (years)		0.27		0.002
severity of depressive symptoms (according to BDI)		-0.23		0.008
Powerful others health locus of control (according to MHLC)		0.12		0.133

Annotations. ^a^ reference group - subjects with a low assessment of quality of life (n = 64)

## Discussion

In the study group of patients with AD, the mean scores of the overall health behaviour index were within the range of average scores according to the Polish norms [32]. Of the three levels of behaviours (low, moderate, high), the low level was the most frequent (40%). Compared to those of healthy controls, the health behaviours of AD patients were significantly lower in terms of the overall health behaviour index and sten scores, which indicated that the proportion of low-level behaviours was more than twice as high in this group. These findings are supported by several previous reports of worse health behaviours in patients with severe mental illness, including affective and other mental disorders, compared to general reference populations, healthy controls and recommended standards [23, 39–41].

The health behaviours of the patients (in terms of overall mean index and standard normalised values) were also significantly worse than those of patients with T2D. Here, the difference was even more marked than that in healthy subjects, as the overall HB index was almost 10 points lower, and the proportion of persons with low health behaviours more than doubled. These results are consistent with previous studies comparing the health behaviour of patients with type 2 diabetes and patients with schizophrenia with co-occurring diabetes [42] and studies analysing the health behaviours of people with diabetes and comorbid severe mental illnesses [43], among others. A possible explanation for the difference found is the unique challenges posed by the nature of the two conditions. In patients with AD, long-term or lifelong periodic mood dysregulation may be accompanied by appetite changes, alteration in food preferences, energy fluctuation, or daily life stressors [44, 45], which in turn can affect their ability to practice and maintain healthy habits. Barriers such as restricted access to high-quality primary and preventive health care, education on targeted lifestyle factors, and economic insecurity might also play a role [40, 46–48]. It is worth emphasising that most studied patients did not receive income from regular employment. All diabetes patients in this study received specialised diabetes care. Therefore, it can be assumed that they received appropriate health education and regular check-ups.

Aspects associated with a positive mental attitude (PMA), i.e., positive thinking, avoidance of negative emotions (anger, anxiety), and maintenance of good relationships within the family contributed the most to variation in the overall HB index. In this respect, the differences in mean scores between the AD group and the comparison groups were the largest and statistically significant. The results obtained are consistent with those of Parletta et al. [23], who reported that compared to people without psychiatric disorders, persons with psychiatric disorders exhibited fewer behaviours contributing to well-being and mentioned that more areas of life generated problems and difficulties. The reasons for the differences between the groups might be the symptoms of mental disorders. The symptoms of AD, in the exacerbated, residual, or subclinical phases, can distort the perception of reality, including one’s own competence, self-efficacy and health behaviours, due to a negative mindset. However, through psychosocial rehabilitation programmes, it is possible to make cognitively distorted self-perceptions more realistic, improve motivation, and increase optimism and self-efficacy in terms of health behaviours, including the mental attitude dimension [49, 50].

Proper dietary habits, including issues of dietary care and the type of food consumed (e.g., whole-grain bread, preserved nutrition), is the second HB category that most differentiated the AD group from the comparison groups, although at the limit of statistical significance (p = 0.078). The results obtained confirm reports by other authors that AD patients, compared to healthy individuals, are characterised by inadequate overall dietary practices and a worse diet in some qualitative respects [51–54]. As highlighted by previous researchers, the dietary pattern of persons with AD, especially those with bipolar disorder, is the result not only of nutritional patterns shaped by various external factors and unfavourable to a healthy lifestyle but also of the complex internal interactions of many neuroendocrine/biological, psychological, and behavioural characteristics specific to AD [54–56].

In the other two health behaviour categories assessed in this study (preventive behaviour and health practices), the mean scores in the AD group were also lower than those in the comparison groups, but the differences were small and nonsignificant.

Among potential sociodemographic, clinical and subjective determinants of health behaviour, overall subjective quality of life, age, and severity of depressive symptoms were found to be independent predictors.

A significant association between health behaviours and the psychological well-being of persons with mental disorders has also been reported by other authors, not only in observational studies but also in interventional studies [57, 58]. However, it should be emphasised that there are numerous reports of no effect or a counterproductive effect. This may occur when awareness of the risks of unhealthy lifestyles increases and there is little opportunity to change existing behaviours to healthy ones and when the objectively desired health behaviours are mismatched and/or not accepted by patients [59, 60].

The positive effect of age on health behaviours demonstrated in the present study corresponds with the results of other research reporting that the practice of certain health-promoting behaviours increases with age, such as eating a healthier diet, reducing smoking and scheduling more frequent health checks [61–63]. The reasons for this regularity are attributed to the necessary limitations resulting from the more frequent appearance of a wide variety of ailments and diseases with ageing, regardless of the presence or absence of a mental illness [62]. Further research is needed to more fully explain the age-related result obtained, especially as comprehensive analyses are lacking in the literature.

Contrary to previous research suggesting that women have a higher propensity to engage in health-promoting behaviours than men [64], the present study did not identify statistically significant gender differences within the examined AD sample. One possible explanation may be that the course of the disease, particularly depression, tends to be more severe in women and is more frequently associated with greater functional impairment and burden, which could diminish or eliminate gender differences in health behaviours [65, 66]. Another explanation for the lack of gender differences may be that health-related behaviours are shaped by multiple factors, including family status, education level, employment status, social support, income, and cultural factors [67]. In this study, the number of potential determinants was limited, and their random arrangement might have led to the inability to detect a significant influence of gender.

The last significant determinant of overall health behaviour identified in the study group was depressive symptoms. As indicated in the literature, the relationship between these two factors is bidirectional, meaning that the presence of depressive symptoms can lead to worsening of health behaviours (e.g., those related to nutrition, physical activity, and sleep), and, conversely, that improving health behaviours can reduce the severity of depressive symptoms [28, 68, 69]. Given these findings and the information presented above, it seems reasonable to assume that targeted therapeutic interventions that, in addition to standard pharmacological treatment and psychological support, include tailored modification of health behaviours, can improve outcomes for patients with AD in terms of both their mental and somatic health. Nevertheless, further research is needed, especially experimental research aimed at confirming the clinical effects on health-promoting behaviours.

### Limitations

This study is one of the few in the literature to analyse the health behaviours of patients with AD and compare them with groups of persons with chronic somatic illness and healthy individuals, enabling us to highlight the specificity of health behaviours of individuals with severe mental disorders. However, this study is not without several limitations. The most important of these are the self-reported data on the health behaviours of the study participants and the nonprobabilistic sampling method. The first is a potential source of bias in the results obtained compared to possible data based on objective measurements. The second warrants caution in generalising the results to the entire population. Another limitation is that the comparative analyses did not include all participants with AD but only a subgroup, which was determined with the goal of making the AD group more comparable to the reference groups. Finally, the last limitation is the lack of measurement of depressive symptomatology in the T2DM and HC groups, which could have provided valuable additional information regarding the inclusion criteria for study participants.

## Conclusions

The results of this study showed that the health behaviours of patients with AD were worse than those of patients with T2D and healthy individuals, mainly regarding positive mental attitudes and eating habits. Lower levels of health behaviours of patients with AD were associated with negative assessment of quality of life, younger age, and more severe depressive symptoms. The findings indicate that a systematic assessment of single and multiple health behaviours that takes into account clinical and sociodemographic factors is needed to identify possible risks for mental and somatic health and to enable the development of better preventive measures and educational programmes for people with mental illnesses.

## Data Availability

The datasets used and/or analysed during the current study available from the corresponding author on reasonable request.

## Abbreviations

AD: Affective disorders
HB: Health behaviours
T2D: Type 2 diabetes
BP: Bipolar disorder
UAD: Unipolar disorder
DALY: Disability-adjusted life years
HBI: The Health Behaviour Inventory
HE: Healthy eating
PB: Preventive behaviours
PMA: Positive mental attitude
HP: Health practices
BDI: Beck Depression Inventory
GAF: Global Assessment Functioning scale
MHLC-B: Multidimensional Health Locus of Control scale
I: Internal locus of control
P: Powerful others
C: Chance
SD: Standard deviation
